# Gut *Akkerm*ansia enhances liver protection and facilitates copper removal during D-penicillamine treatment in a Wilson’s disease model

**DOI:** 10.1128/spectrum.00573-24

**Published:** 2025-03-31

**Authors:** Xi Huang, Yanqi Jin, Tianyuan Wang, Danting Fu, Jindi Ma, Xiaopeng Yu, Yingfeng Lu, Jingyuan Song, Yu Chen, Ren Yan, Yimin Zhang

**Affiliations:** 1 Department of Electrocardiogram, The First Affiliated Hospital, School of Medicine, Zhejiang University, Hangzhou, Zhejiang, China; 2 State Key Laboratory for Diagnosis and Treatment of Infectious Diseases, National Clinical Research Center for Infectious Diseases, Collaborative Innovation Center for Diagnosis and Treatment of Infectious Diseases, The First Affiliated Hospital, College of Medicine, Zhejiang University, Hangzhou, Zhejiang, China; 3 Department of Hospital-Acquired Infection Control, Affiliated Hangzhou First People’s Hospital, Zhejiang University School of Medicine, Hangzhou, Zhejiang, China; 4 Department of Experimental Animals, Zhejiang Academy of Traditional Chinese Medicine, Hangzhou, Zhejiang, China; 5 Department of Infectious Diseases, Haining People’s Hospital, Haining, Zhejiang, China; University of California, Davis, San Bernardino, California, USA

**Keywords:** copper toxicity, liver injury, gut microbiota, pharmacomicrobiomics, *Akkermansia muciniphila*, copper removing, Wilson’s disease

## Abstract

Patients afflicted with Wilson’s disease (WD) may encounter hepatic and extraneous manifestations due to the progressive accumulation of copper in the liver and other subsequent organs. Copper-chelating agents, such as D-penicillamine (DPA), are commonly utilized in the medical treatment of copper overload in WD. Manipulating the composition of gut microbiota appropriately can enhance drug efficacy and safety. This study aims to investigate how targeted intervention on gut microbiota influences the effectiveness of copper removal in a WD model during DPA treatment. First, following a 4-week treatment of DPA, the liver copper concentration and gut microbial composition were assessed in the WD mice model to identify potential candidates for targeted regulation of gut microbiota. Second, after 8 weeks of manipulating the gut microbiota during DPA treatment, various parameters including blood liver function indicators, tissue copper load, hepatic histopathological features, and gut microbiota were investigated in WD mice. The findings demonstrated that the presence of *Akkermansia* significantly enhances the efficacy of DPA, leading to a more efficient elimination of copper from tissues and a greater improvement in liver injury, liver dysfunction, and gut dysbiosis. In contrast, *Butyricimonas* has an antagonistic effect. The results of gene function prediction analysis indicated that the altered gut microbial function by DPA and Akk is primarily linked to energy generation/utilization, amino acid, fatty acid, lipid, and nucleic acid metabolisms. To summarize, this study provides experimental evidence for the potential application of targeted regulation of gut microbiota in the adjunctive therapy of copper dysregulation disease.

**IMPORTANCE:**

Copper is an essential element in virtually all living organisms. Wilson’s disease (WD) is a representative disorder caused by the disruption of copper homeostasis. Oral-chelating agents are the first-line treatment for copper-overloaded diseases, with D-penicillamine (DPA) being the prototypical drug. However, the efficacy and adverse effects of DPA remain challenging in its use for WD treatment. In our study, the supplementation of *Akkermansia muciniphila* (Akk), a key gut microbe, along with DPA was demonstrated to enhance copper removal, ameliorate liver injury and dysfunction, and restore gut dysbiosis in a mouse model of WD. These findings highlight the significant potential applications of targeted modulation of gut microbiota as “pharmacomicrobiomics” in adjunctive therapy for disorders involving copper dysregulation.

## INTRODUCTION

Copper, an essential element of life, plays a pivotal role as a crucial catalytic cofactor in diverse biological processes, ranging from bacteria and fungi to plants and animals. These processes encompass mitochondrial respiration, antioxidant defense, and the synthesis of bio-compounds (1). In regular circumstances, the cellular copper level is actively regulated through a sophisticated homeostatic mechanism to maintain an extremely low level, thereby preventing excessive copper accumulation that could potentially harm the cell (2). However, genetic variation of copper homeostasis can give rise to copper dysregulation syndromes, such as Menke’s disease (MD), characterized by copper deficiency, and Wilson’s disease (WD), characterized by copper overload. The mutation of the *ATP7B* gene disrupts the excretion of copper in patients with Wilson’s disease, resulting in the progressive accumulation of copper in the liver, brain, and other tissues (3). The initial clinical manifestation of WD often involves hepatic dysfunction with symptoms including liver failure, elevated liver enzyme levels, jaundice, chronic hepatitis, and potentially cirrhosis (4). Furthermore, DNA damage, lipid peroxidation, and mitochondrial dysfunction are also commonly observed in the liver of WD patients (5). Medical treatment of copper overload in WD includes the use of zinc salts to reduce copper absorption in the gastrointestinal tract. Additionally, oral chelators like D-penicillamine (DPA), trientine, and dimercaprol are employed for medical management (6). DPA and trientine are generally considered safe and effective as primary oral chelators for treating WD (7).

The composition and biodiversity of gut microbiota have been found to be altered in patients with WD (8), whereas copper exposure has also been reported to induce liver damage and gut dysbiosis during early life (9). The significance of the gut microbiota in maintaining good health and contributing to the development of diseases is widely acknowledged. Moreover, the gut microbiota plays a crucial role in drug absorption and availability, making it an attractive approach for enhancing medication effectiveness and safety (10). Prolonged use of copper chelators is associated with various adverse events (AEs), including gastrointestinal symptoms and immune disorders (11, 12). Specifically regulating the gut microbiota has been demonstrated to be an effective strategy for enhancing both gastrointestinal and systemic immune function (13). For instance, the probiotic *Bacillus subtilis* BS50 has shown potential in reducing gastrointestinal symptoms (14), whereas the administration of a combination probiotic drug CBLEB (Combination of *Bifidobacterium*, *Lactobacillus*, *Enterococcus*, and *Bacillus*) has exhibited a therapeutic effect on immunodeficiency (15). On the other hand, the composition of gut microbiota is influenced by various factors, including medication usage. It has been reported that approximately 10% of inter-individual variation in the gut microbiome can be attributed to medication use (16). Not only antibiotics but also non-antibiotic drugs can disrupt gut microbial balance and indirectly lead to tissue injury (17–19).

Therefore, the objective of this study was to investigate the alterations of gut microbiota in simulated WD mice with copper overloading during DPA treatment and subsequently explore the impact of targeted modification of gut microbial composition on the therapeutic efficacy of copper removal.

## RESULTS

### DPA-altered gut microbes are closely correlated to its liver copper-removing effect on WD mice

**Fig 1:**
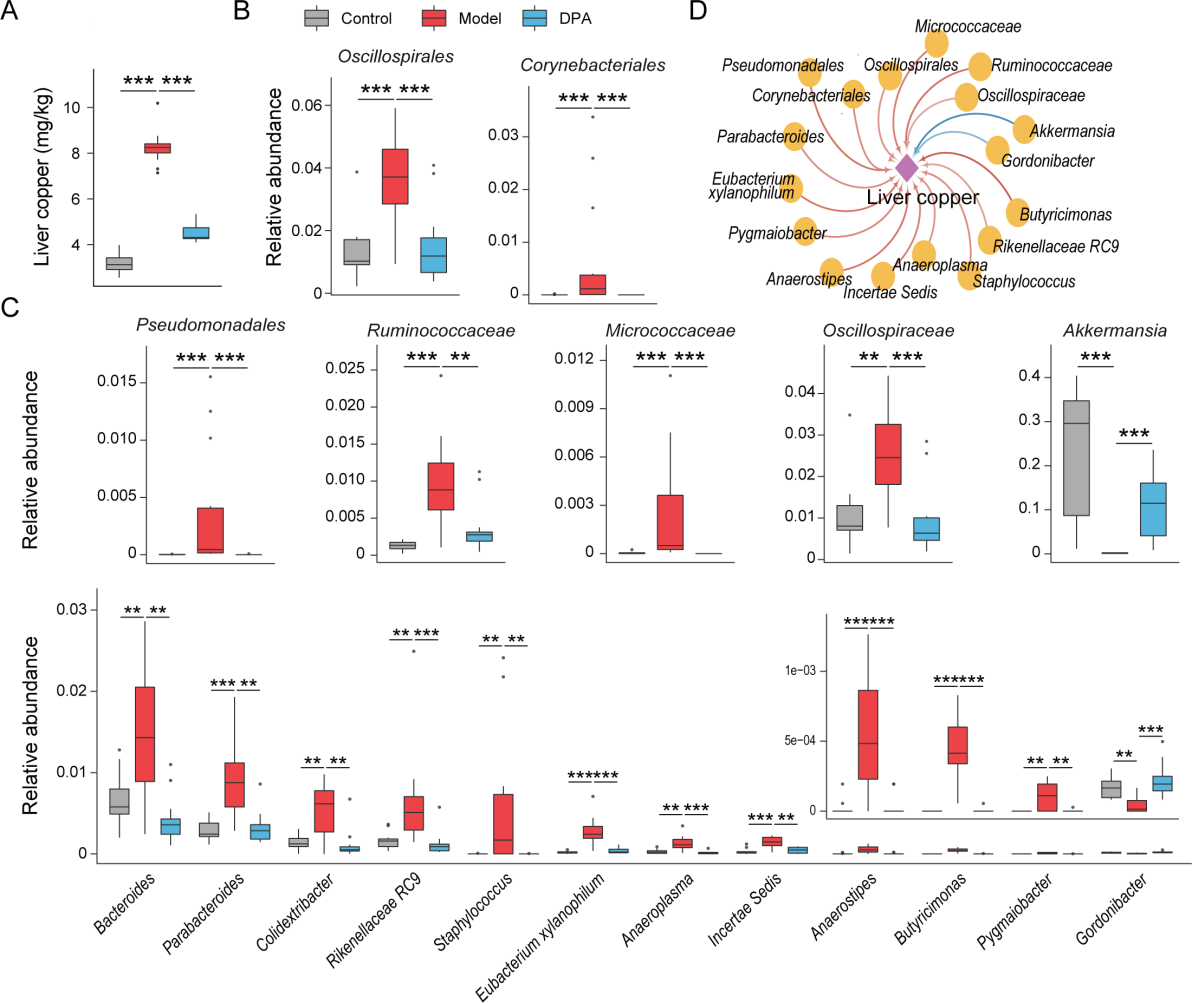
The liver copper-removing effect of DPA is related to its regulation in gut microbiota of WD mice. The liver copper concentration (**A**) and relative abundance of some gut bacterial taxa (**B and C**) were recovered after 4 weeks of DPA treatment. (**D**) Correlation of DPA-influenced gut microbes with liver copper. *P < 0.05, **P < 0.01, ***P < 0.001

During the first stage of the experiment, we examined the impact of copper overload on the composition of a healthy gut microbiota and evaluated the influence of oral DPA supplementation on gut microbiota under conditions of copper overload. The liver and fecal samples were collected from groups A, B, and C at the end of the eighth week for 16 s rDNA amplicon sequencing. It was observed that the elevated concentration of liver copper in WD mice significantly improved after 4 weeks of DPA treatment (Fig. 1A). At the same time, the gut dysbiosis in WD mice was partially reversed by DPA. The increased gut bacterial taxa, including *Oscillospirales*, *Corynebacteriales*, *Pseudomonadales*, *Ruminococcaceae*, *Micrococcaceae*, *Oscillospiraceae*, *Bacteroides*, etc., and the depleted taxa *Akkermansia* and *Gordonibacter* returned to normal levels after DPA treatment (Fig. 1B and C). In order to investigate microorganisms potentially associated with the copper removal effect of DPA, we performed the Spearman correlation test between liver copper levels and these differential taxa. The results showed that the bacterial taxa *Akkermansia* (r = −0.787, *P* = 1.03E-9) and *Butyricimonas* (r = 0.803, *P* = 2.54E-10) have the most closest relationship with liver copper (Fig. 1D). This finding indicates that *Akkermansia* may have a synergistic effect on DPA in the treatment of WD mice, whereas *Butyricimonas* may play an antagonistic role.

### Akk administration promotes copper removal and improves liver injury during the DPA treatment on WD mice

**Fig 2:**
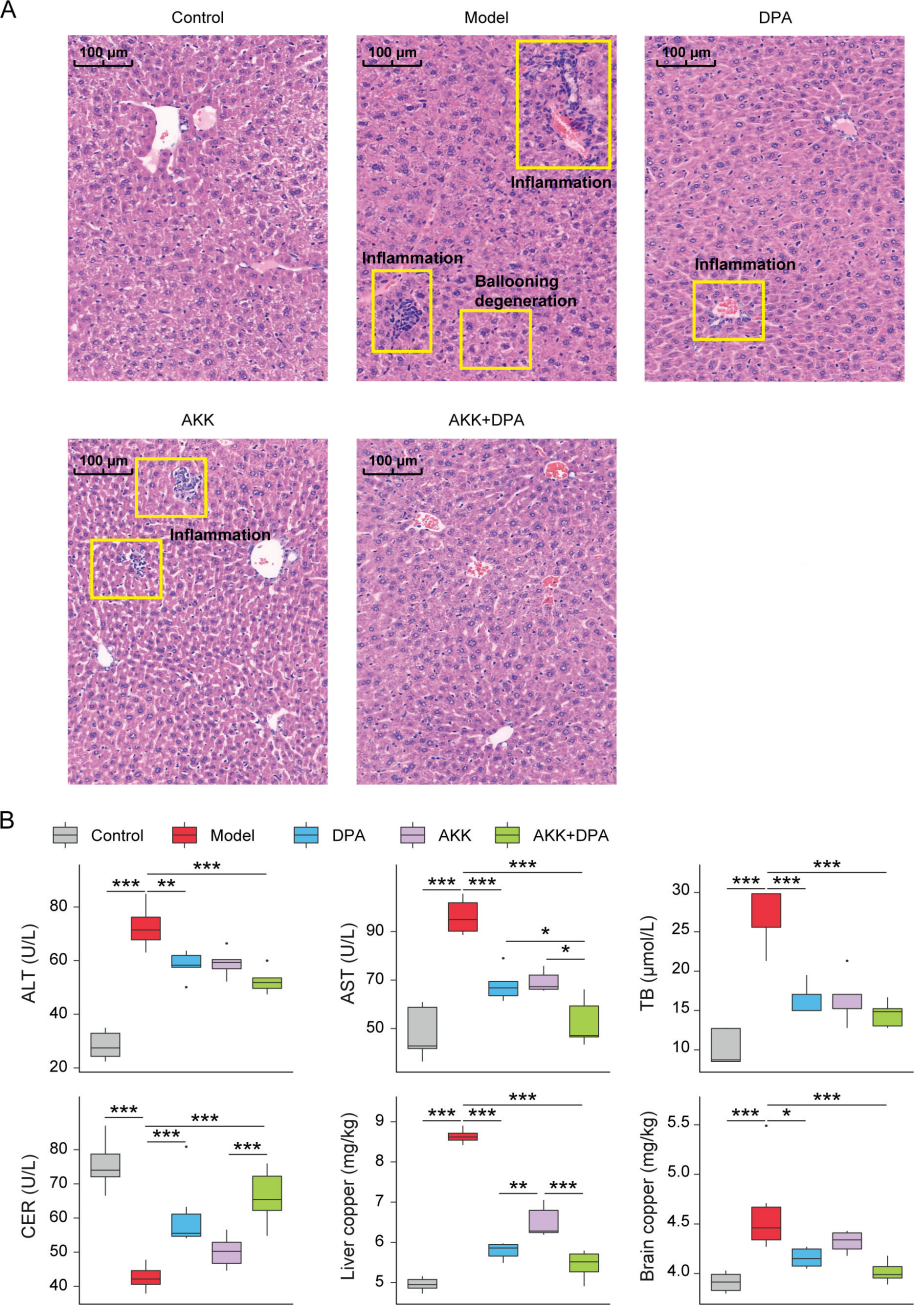
Akk administration improved copper-removing efficacy in WD mice. (**A**) Representative images of liver samples stained by HE. (**B**) Levels of serum ALT, AST, TB, CER, and copper concentration in liver and brain. *P < 0.05, **P < 0.01, ***P < 0.001.

To validate the correlation analysis result, in the second stage of the experiment, we investigated the impact of *Akkermansia* administration on DPA treatment. The standard strain Akk was selected as a representative. After 8 weeks of DPA, Akk, or Akk + DPA treatment, we analyzed liver histopathology, liver function indicators, tissue copper load, and gut microbiota of WD mice. The photomicrographs obtained from H&E staining revealed significant cellular ballooning degeneration and inflammatory infiltration in the model group (Fig. 2A). In the DPA and Akk + DPA groups, there was a tendency toward normal hepatocyte morphology with reduced cellular ballooning degeneration and inflammatory infiltration. Furthermore, compared with DPA alone, Akk + DPA exhibited a more pronounced protective effect on liver injury, as evidenced by the absence of inflammatory cells.

The results of our study indicated that DPA, Akk, and their combinations exhibit varying degrees of efficacy in improving liver function and tissue copper concentration in WD mice (Fig. 2B). First, both DPA and Akk + DPA significantly alleviated the elevation of serum ALT, AST, and TB levels, as well as liver and brain copper concentrations while mitigating the reduction in serum ceruloplasmin (CER) level observed in WD mice. Second, compared with DPA alone, Akk + DPA treatment exhibited a more pronounced effect on reducing serum AST level. Furthermore, although Akk treatment alone did not yield significant improvements in the aforementioned indicators, a similar trend was observed. These findings confirm the synergistic effect of Akk when combined with DPA for treating WD mice.

**Fig 3:**
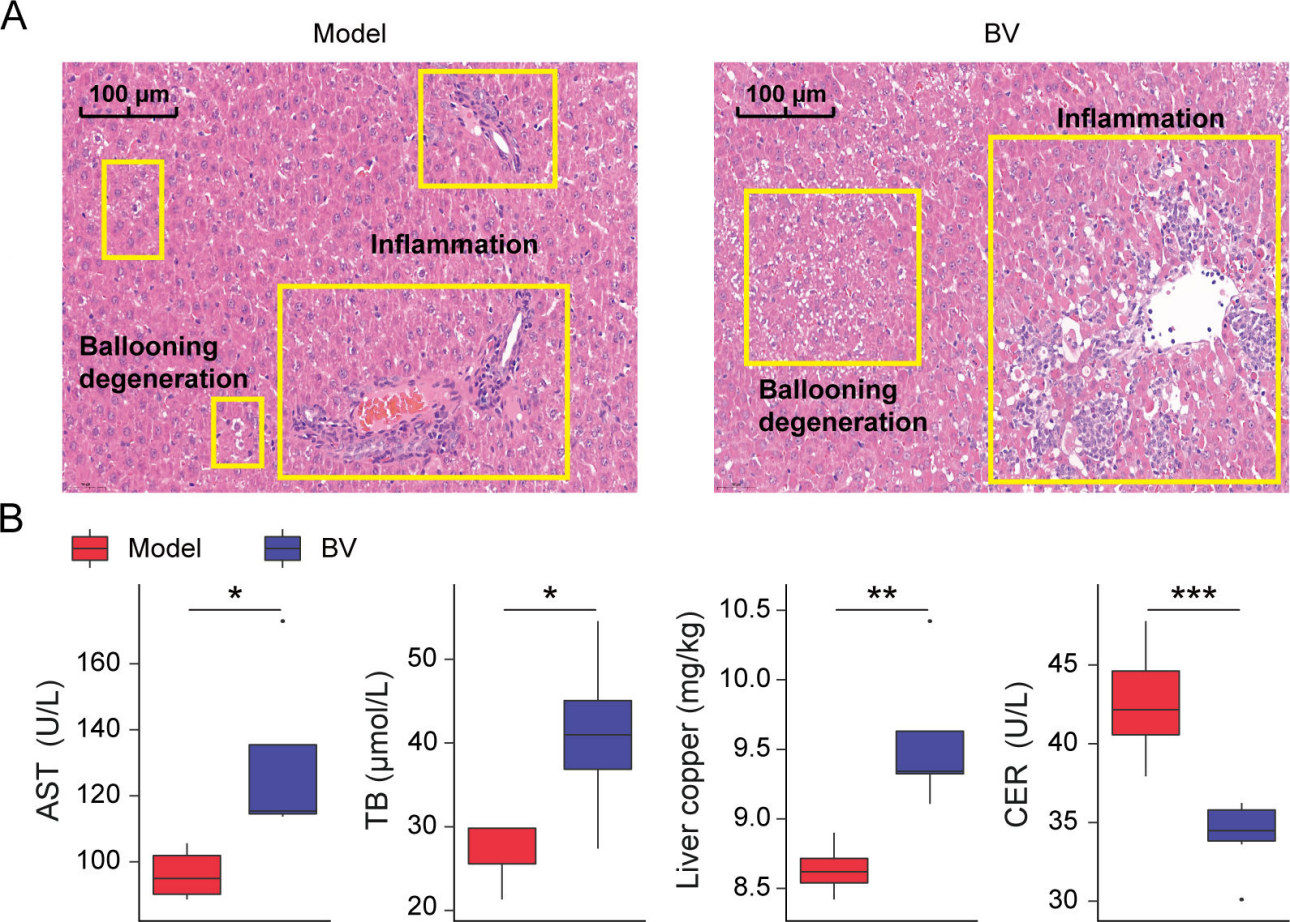
BV administration aggravated the injury of copper poisoning in WD mice. (**A**) Representative images of liver samples stained by HE. (**B**) Levels of serum AST, TB, CER, and liver copper concentration. *P < 0.05, **P < 0.01, ***P < 0.001.

Next, we verified the role of *Butyricmona* in WD mice by selecting the standard strain BV as a representative. We observed that WD mice exhibited more pronounced liver injury phenotypes, such as hepatocyte ballooning degeneration and inflammatory infiltration (Fig. 3A), along with elevated serum AST and TB levels, liver copper concentration, and decreased CER level (Fig. 3A), following an 8-week administration of BV. Therefore, our findings substantiated that BV exacerbates liver injury and dysfunction in WD mice.

### The combined AKK and DPA treatment is more helpful for the recovery of gut dysbiosis in WD mice

**Fig 4:**
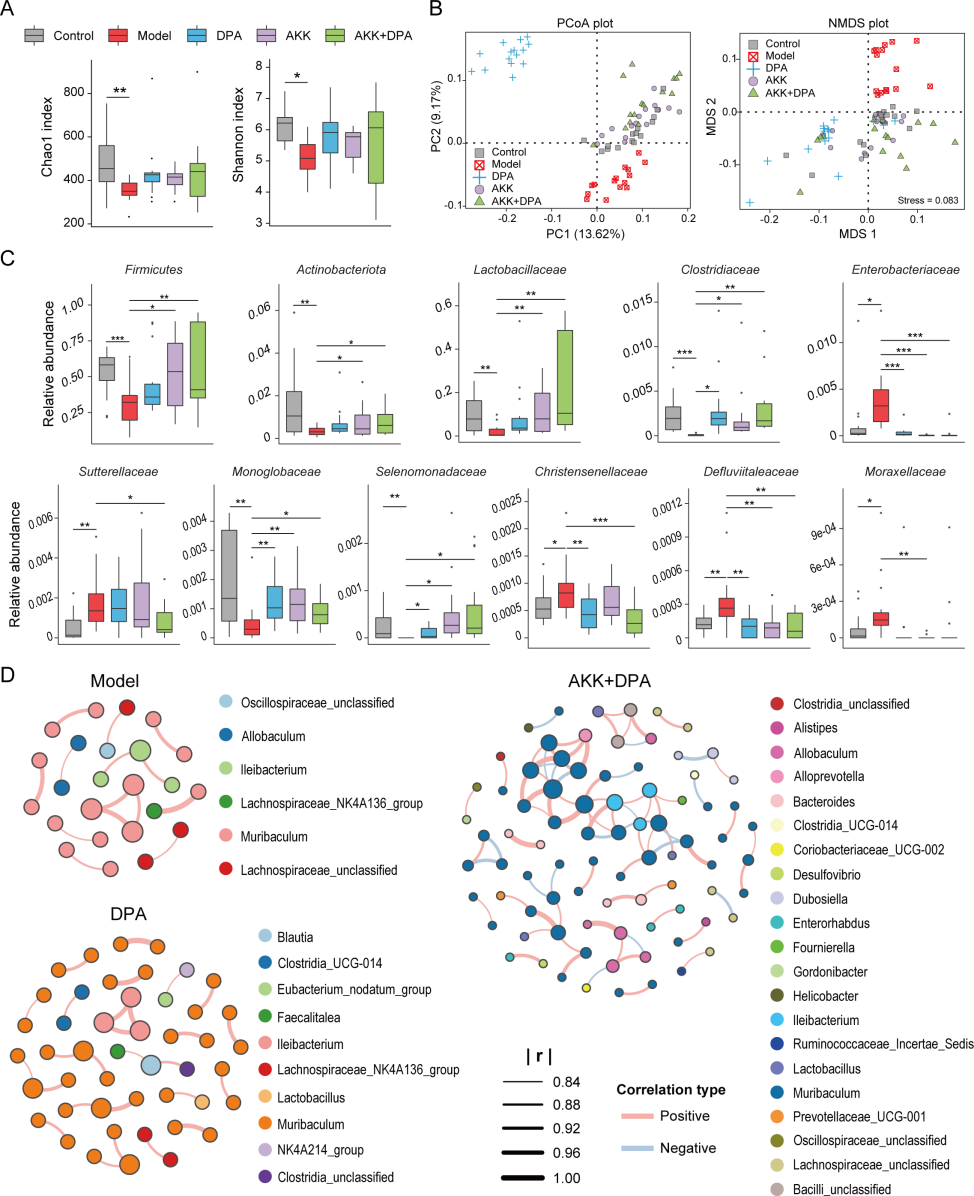
DPA and Akk treatment alleviates gut dysbiosis in WD mice. (**A**) Box plot of richness and community biodiversity estimated based on the Chao1 and Shannon indices. (**B**) Two-dimensional PCoA and NMDS plots based on the unweighted unifrac dissimilarity. (**C**) Alterations in the relative abundance of bacterial taxa in the control, model, DPA, Akk, and Akk + DPA groups. (**D**) Microbial network analysis of model, DPA, and Akk + DPA groups. | R | >0.7 and *P*_adj_ <0.001 were used as significance thresholds. *P < 0.05, **P < 0.01, ***P < 0.001.

The impact of Akk and DPA on the gut microbiota was assessed by conducting a quantitative analysis of the gut bacteria based on 16S rDNA amplicon sequencing. The Chao 1 and Shannon indices revealed significant disparities in community richness and biodiversity between the control and model groups. However, no significant differences were observed among any other pair of two groups (Fig. 4A). Additionally, beta diversity among the five groups of WD mice was visualized through principal coordinate analysis (PCoA) and nonmetric multidimensional scaling (NMDS) plots (stress = 0.083) (Fig. 4B), with intergroup differences assessed using Adonis to determine their significance (Pr [>F] = 0.001). These findings indicate notable variations in beta diversity across all comparisons.

We further investigated the variations in the proportion of specific bacterial taxa among the five groups. At phylum and family levels, the depletion of bacterial taxa, such as *Firmicutes*, *Actinobacteriota*, *Lactobacillaceae*, *Clostridiaceae*, *Monoglobaceae,* and *Selenomonadaceae*, as well as the increase in taxa, such as *Enterobacteriaceae*, *Sutterellaceae*, *Christensenellaceae*, *Defluviitaleaceae,* and *Moraxellaceae* in WD mice, were mitigated following treatment with DPA, Akk, or Akk + DPA treatment compared with the control group (Fig. 4C). It is worth noting that among them, the abundance of five taxa, namely *Firmicutes*, *Actinobacteriota*, *Lactobacillaceae*, *Sutterellaceae,* and *Moraxellaceae*, were recovered only in the treatment groups in which Akk participated (Akk or Akk + DPA groups), but not in the DPA group. Similarly, at the genus level, of the 29 taxa that exhibited a return to normal abundance after treatment, four of them (*Acinetobacter*, *Enterorhabdus*, *Eubacterium ruminantium* group, and DNF00809) demonstrated recovery exclusively in the groups receiving Akk supplementation (Fig. S1). These findings suggest that the administration of Akk can effectively promote the restoration of gut dysbiosis in WD mice.

In order to obtain a more comprehensive understanding of the interrelationships between gut microorganisms influenced by DPA and AKK, we conducted a microbial network analysis on the gut microbiota of the model, DPA, and Akk + DPA groups. The absolute value of the correlation coefficient (r) greater than 0.7 and the adjusted *P* value less than 0.001 were considered the significance threshold. As shown in Fig. 4D, the dominance of *Muribaculum* in the core microbial network of all three groups signifies its pivotal role within the gut microbiota influenced by copper overload. The treatments of both DPA and Akk + DPA can enhance the complexity and diversity of the gut microbial network affected by copper overload, indicating their potential to ameliorate the impaired structure of gut microbiota. Particularly, after the participation of Akk in copper removal therapy, 21 genera of microbial taxa entered the core microbial network, which was 3.5 times greater than that of the model group and twice as much as that of the DPA group.

### The main association of DPA and Akk-altered gut microbial genes is with the generation and utilization of energy, as well as the metabolism of amino acids and fatty acids

**Fig 5:**
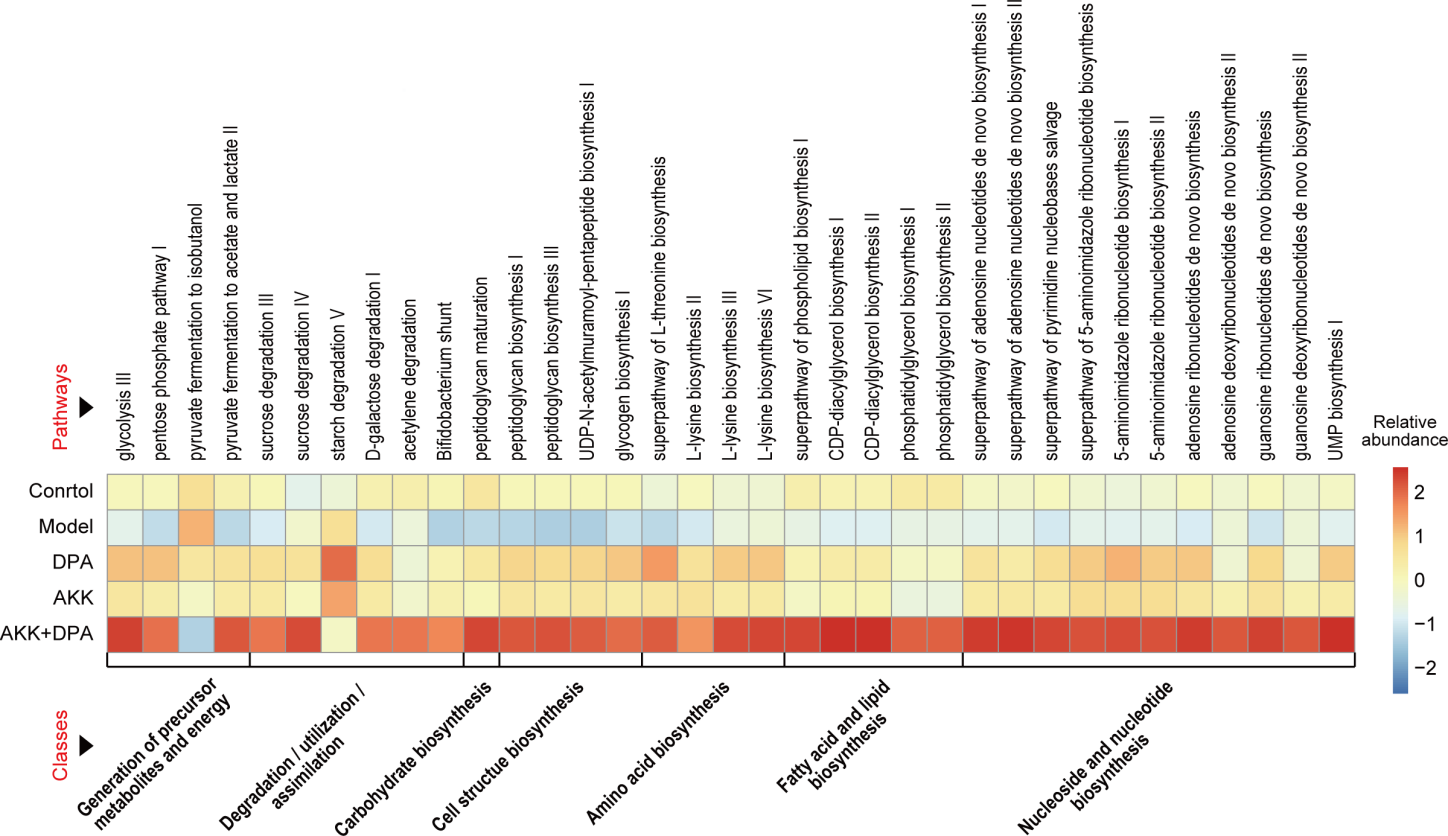
Pathway enrichment of differential gut microbial genes between the control, model, DPA, Akk, and Akk + DPA groups based on function prediction using Picrust 2 (*P* < 0.05).

To investigate the potential role of DPA and Akk-altered gut microbes, we utilized Picrust 2 software to predict the functionalities of distinct gut microbial genes among the control, model, DPA, Akk, and Akk + DPA groups. Subsequently, we aligned these genes with the MetaCyc pathway database and categorized them based on their abundance. The results presented in Fig. 5 demonstrate that these functional genes were predominantly concentrated within seven categories: generation of precursor metabolites and energy, degradation/utilization/assimilation, carbohydrate biosynthesis, cell structure biosynthesis, amino acid biosynthesis, fatty acid and lipid biosynthesis, and nucleoside and nucleotide biosynthesis (*P* < 0.05). It is evident that the combined treatment of Akk + DPA yielded the most significant disparity in functional enrichment among the groups.

**Fig 6:**
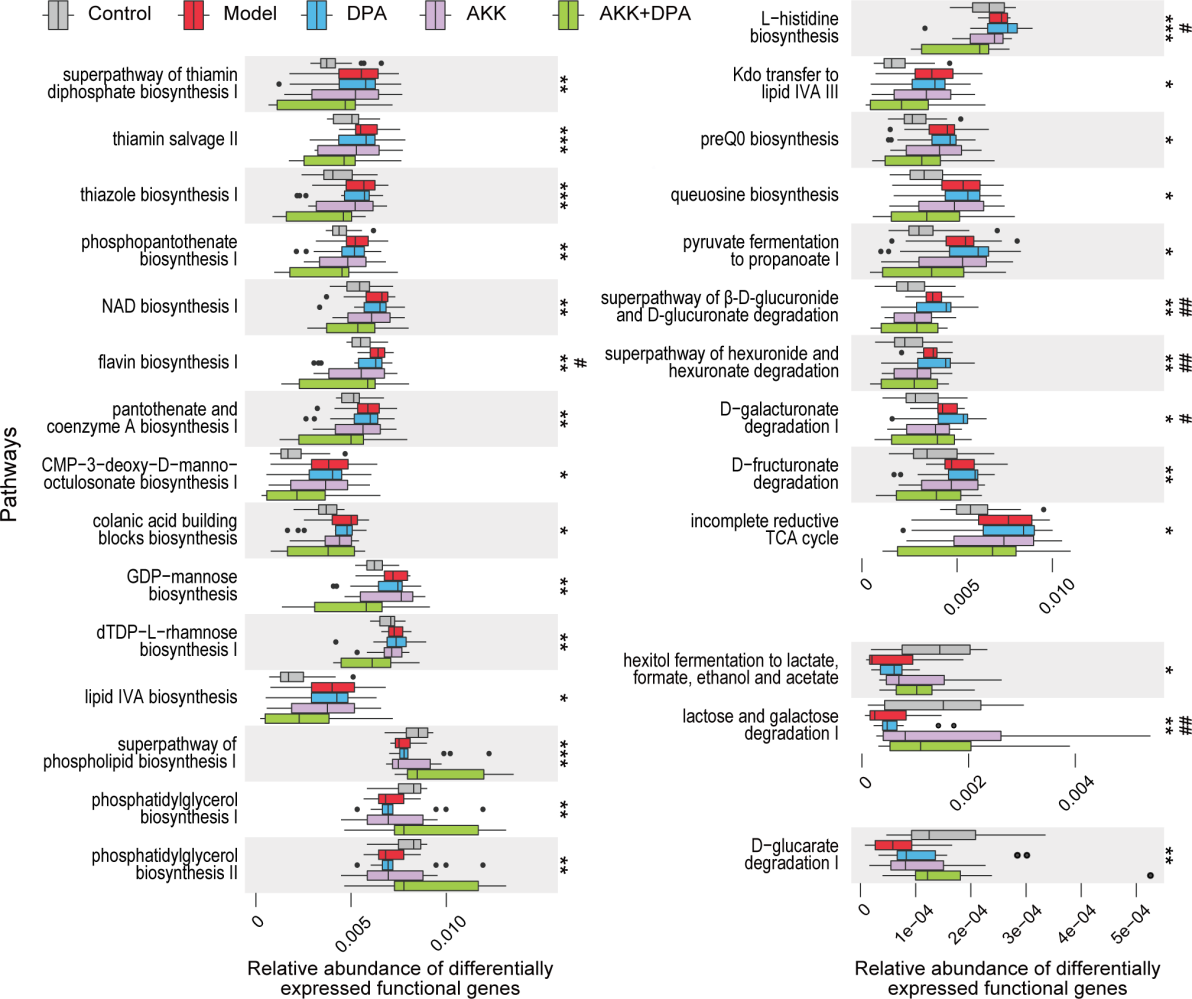
Pathways of gut microbial genes altered in WD mice and whose alterations were alleviated by Akk or Akk + DPA treatment. *P < 0.05, **P < 0.01, ***P < 0.001 in model vs. Akk +DPA. # *P* < 0.05, ## *P* < 0.01 in model vs. Akk.

Afterward, we conducted a more in-depth analysis of the functional variations among the distinctively enriched gut microbes in each group. To investigate the regulatory effect of Akk on the gut microbial function of WD mice, we screened for abnormal functional pathways that were only recovered in the Akk participated groups. As a result, 22 WD-upregulated genes located in pathways and 6 WD-downregulated genes located in pathways were restored after treatment with Akk + DPA, whereas six pathways were restored after treatment with Akk alone (Fig. 6).

## DISCUSSION

Copper is an essential element in nearly all living organisms, and maintaining systemic copper levels within a specific range is crucial for normal biochemical processes (1). Imbalances in copper homeostasis have been associated with various pathological conditions, including MD, WD, neurodegenerative disorders, cancer, and cardiovascular diseases. Excess copper ions can induce cuproptosis, a recently identified form of regulated cell death (2), which is believed to contribute to the progression of WD and may serve as a potential therapeutic target for this disease (1). The first-line treatment for copper overload is oral chelating agents, which are generally safe and effective. However, prolonged use of these agents can still result in various AEs including gastrointestinal symptoms and immune disorders. There exists a complex relationship between orally ingested drugs and gut microbes, as drugs have the ability to modify the composition and function of gut microbes, whereas gut microbes can actively participate in the chemical conversion of drugs. The concept of “pharmacomicrobiomics” has garnered attention in recent years due to an increasing body of evidence demonstrating the influence of gut microbiota on drug bioavailability, bioactivity, and toxicity (10, 20). Furthermore, rectifying gut dysbiosis under pathological conditions has proven to be an effective adjuvant treatment (21–24). In this study, we investigated the alterations in the gut microbiota in a copper overload WD model treated with DPA and aimed to enhance therapeutic efficacy by manipulating the composition of gut microbes.

We assessed the liver copper concentration and gut microbial composition in WD mice after a 4-week treatment with DPA. Through correlation analysis, we identified *Akkermansia* and *Butyricimonas* as the bacterial taxa most strongly associated with changes in liver copper concentration, although their roles may be diametrically opposed. Our validation study confirmed that supplementation with Akk had superior therapeutic effects compared with DPA treatment alone, including reduced liver damage, decreased liver and brain copper concentration, lowered levels of ALT, AST, and TB, as well as increased CER level. Conversely, administration with BV exacerbated the liver pathological phenotype in WD mice to a greater extent, leading to increased liver copper concentration and elevated levels of AST and TB, whereas CER level decreased. *Akkermania*, especially its representative strain Akk, is widely regarded as the next generation of probiotics and biotherapeutic agents due to their profound health-promoting effects on a variety of diseases and physiological dysfunctions. As a result, it has emerged as a prominent global trend and research focus in the medical field (25–27). The impaired ATP7B function in Wilson’s disease results in the failure to incorporate copper into CER and excrete copper through bile, leading to the accumulation of copper in the liver and subsequent development of chronic liver disease and cirrhosis (28). The supplementation of Akk has been extensively reported to improve a range of liver diseases, encompassing both alcoholic and nonalcoholic, as well as drug-induced liver injury, by modulating the gut-liver axis (29–32). Although there have been some contentious reports in recent years regarding the health-promoting effects of Akk, suggesting its potential to exacerbate certain conditions such as colitis and multiple sclerosis (particularly in cases of gut immunometabolic disorders) (33–37), our findings still affirm that Akk exerts a hepatoprotective effect against copper toxicity-induced liver damage. On the other hand, *Butyricimonas* species are generally regarded as beneficial gut microbes due to their ability to produce butyric acid (38). For instance, supplementation with BV has been shown to prevent obesity induced by a high-fat diet (39). However, certain *Butyricimonas* species such as BV and *Butyricimonas faecihominis* have been associated with bacteremia, bloodstream infection, and bowel disease in some cases (40–43). Consistent with our findings, a study reported that *Butyricimonas* was positively correlated with hepatic inflammation and oxidative stress markers while *Akkermania* exhibited the opposite effect in an alcohol-induced liver damage mouse model treated with vinegar extract (44).

Subsequently, we conducted further investigations into the alterations in gut microbial composition, networks, and function in WD mice treated with DPA, both with and without Akk supplementation, aiming to explore its probiotic properties and possible mechanisms. Our findings indicated that Akk supplementation is beneficial for the recovery of gut dysbiosis in WD mice. First, we observed a decrease in conditionally pathogenic bacteria such as *Enterobacteriaceae* and an increase in beneficial bacteria such as *Lactobacillaceae* only in the groups receiving Akk supplementation. Patients with WD also exhibited a significantly higher prevalence of *Enterobacteriaceae*, which may be associated with compromised intestinal immune and metabolic functions in these individuals (8). It is noteworthy that the relative abundance of *Lactobacillaceae* and *Lactobacillus* exhibited a significant increase following Akk supplementation. Moreover, network analysis has confirmed that Akk supplementation enhanced the complexity and diversity of the gut microbial network affected by copper overload, with *Lactobacillus* being integrated into the core network (| r | >0.7, and *P*_adj_ <0.001). Several lactic acid bacteria, such as *Lactobacillus helveticus* CD6, *Lactobacillus casei* KCTC 3260, and *Streptococcus thermophilus* 821, known for their antioxidant properties, have been reported to possess strong chelating ability toward ferrous and copper ions (45–47). Therefore, although there is no direct evidence of Akk’s ability to directly chelate metal ions, we speculate that alterations in gut microbial composition induced by Akk may enhance intestinal copper chelation capacity in WD mice. Second, by utilizing functional prediction, we found that the gut microbial glycolysis III pathway (from glucose) as well as the pyruvate fermentation to acetate and lactate II pathway in WD mice were upregulated due to Akk supplementation. A recent investigation has revealed that copper-dependent death occurs through the direct binding of copper to lipoylated components of the TCA cycle. This binding leads to the aggregation of copper-bound lipoylated mitochondrial protein and subsequent loss of ferrous-sulfur cluster protein, resulting in the development of proteotoxic stress and ultimately leading to cell death (2). According to this theory, it is considered essential for cuproptosis that pyruvate is taken up by mitochondria to initiate the TCA cycle. Therefore, increased glycolysis and inhibitors of pyruvate mitochondrial uptake can attenuate cell death. Thus, we speculate that the gut microbiota regulated by Akk may potentially derive increased energy through glycolysis and subsequent pyruvate fermentation rather than relying on the TCA cycle. This metabolic adaptation could contribute to enhanced resistance against cuproptosis.

In conclusion, this study describes the synergistic effect of key gut microbe Akk on DPA treatment of copper overload WD mice. Indeed, this study lacks a comprehensive exploration of the functional mechanisms of Akk; however, our phenotypic evidence suggests that supplementation of Akk can effectively facilitate copper removal, enhance liver injury and liver dysfunction recovery, and provide greater assistance in restoring gut dysbiosis in WD mice during DPA treatment. These findings imply that the targeted manipulation of gut microbiota holds significant potential in the adjunctive management of copper dysregulation disease.

## MATERIALS AND METHODS

### Microorganisms

*A. muciniphila* BAA-835 and *B. Virosa* JCM15149 were purchased from the American Type Culture Collection (ATCC). Akk was cultured in a modified brain-heart infusion (BHI) liquid medium (Difco, MI, USA) at 37°C under anaerobic conditions for 48 h and harvested by centrifugation at 8,000 × *g* for 10 min at 4°C. BV was cultured in a Columbia broth supplemented with 5% horse serum at 37°C under anaerobic conditions for 72 h and harvested by centrifugation at 8,000 × *g* for 15 min at 4°C. Then, the concentration of the Akk and BV was adjusted to the dose used in the oral gavage.

### Animal experimental design and sample collection

**Fig 7:**
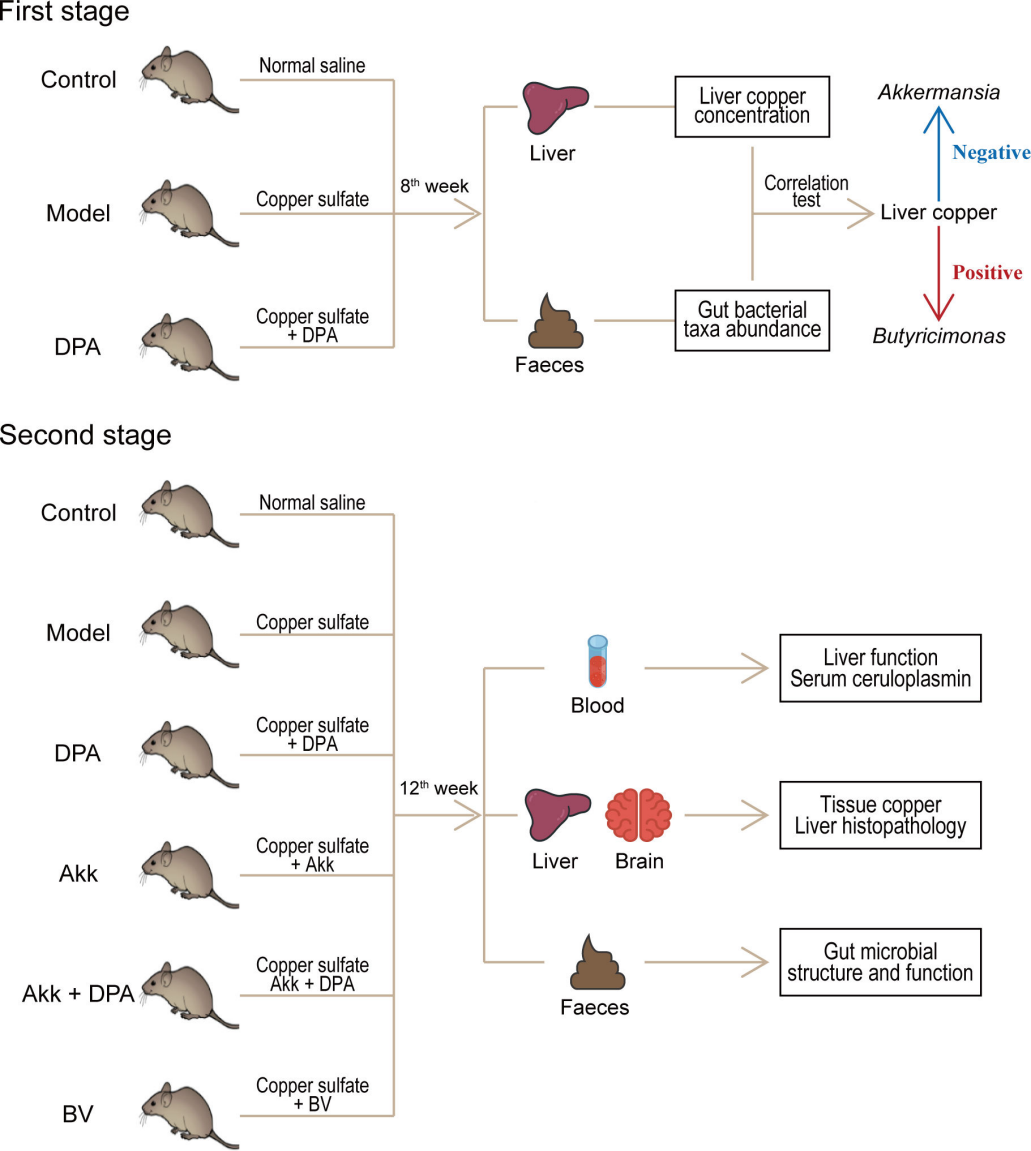
Schematic diagram of the Animal experimental design. NS, normal saline.

Specific pathogen-free male mice (C57BL/6, 8 weeks of age) were obtained from the Zhejiang Weitong Lihua Experimental Animal Technology Company. Prior to the experiment, the mice were acclimatized for a duration of 2 weeks while being provided a regular diet and kept in a room set at room temperature (RT) with a 12/12 h day/night cycle. As shown in Fig. 7, in the first stage, 90 mice were divided into three groups (with 30 mice in each group, 5 mice per cage), namely (i) control, (ii) model, and (iii) DPA treatment; in the second stage, an additional 45 mice were assigned to three groups (with 15 mice in each group, 5 mice per cage), namely (iv) Akk treatment, (v) Akk + DPA treatment, and (vi) BV treatment. All mice except those in group A received 0.185% copper sulfate (0.02 mL/g body weight/day) for 12 weeks (48). Mice in groups C, D, E, and F were orally gavaged with corresponding DPA (0.01 mg/g body weight/day), Akk (3 × 10^9^ CFU/day), BV (3 × 10^9^ CFU/day), or their combination from 5th to 12th weeks. Mice in group A were gavaged with the same volume of normal saline. Fecal samples of the 8th and 12th week were collected for 16 s rDNA amplicon sequencing. At the end of the 8th week, 45 mice in A, B, and C groups (*N* = 15 in each group) were anesthetized with pentobarbital sodium before sacrifice, and liver tissue samples were collected for copper assay. After 12 weeks, the remaining mice were given anesthesia. Samples of liver tissue were preserved in 10% formalin solution for 24 h, followed by embedding in paraffin wax. The tissue was then sliced into sections that were 3 μm thick and subsequently stained using hematoxylin and eosin (H&E). Blood samples were collected and immediately used or stored at −80°C until use. All experimental procedures were approved by the Committee on the Ethics of Animal Experiments of Zhejiang Academy of Traditional Chinese Medicine (approval number: [2021]−037).

### Blood indicator testing and tissue copper assay

Liver function indicators, including alanine aminotransferase (ALT), aspartate aminotransferase (AST), and total bilirubin (TB), were tested using a Fuji DRI-CHEM 4000ie dry chemistry analyzer (Fujifilm, Tokyo, Japan). The level of CER was measured by using the ELISA kit (Fankewei, Shanghai, China).

For liver and brain copper assays, 10 mg of each sample was acid-digested using a mixture of HNO_3_ and HCl in an ETHOS microwave digestion system (Milestone, Shelton, CT, USA). After digestion, the samples were made up to 5 mL using ultra-pure water. The Thermo ICP-MS system (ThermoFisher Scientific, Waltham, MA, USA) was used to measure the amount of copper following the guidelines provided by the manufacturer. The ICP-MS system was operated in a single He KED mode with parameters as: RF power, 1550 W; Pump Speed, 40 rpm; S/C temperature, 2.7°C; Smpl Depth, 5 mm; Cool flow, 14 L/min; Auxiliary flow: 0.8 L/min; and Nebulizer flow, 1.122 L/min.

### Fecal 16s rRNA sequencing and analysis

DNA from fecal samples was extracted using a Qiagen PowerSoil Kit (Qiagen, Hilden, Germany) according to the manufacturer’s protocols. Primers 338F (5′-ACTCCTACGGGAGGCAGCAG-3′) and 806R (5′-GGACTACHVGGGTWTCTAAT-3′) were used to amplify the V3–V4 variable region of 16S rDNA. The AMPure XP beads (Agencourt, Beckman Coulter, United States) were utilized to purify the PCR products, and the library was measured for quantity using RT-qPCR. Sequencing of DNA was carried out using an Illumina NovaSeq system (Illumina, San Diego, CA, USA), and 250 bp paired-end reads were generated. After truncating the barcode and primer sequence, Flash software (v1.2.11) was utilized for merging the reads of each sample to obtain raw tags. The fastp software (v0.20.0) is utilized to obtain high-quality clean tags from raw tags, followed by the use of Vsearch software (v2.15.0) to eliminate chimeras and acquire effective tags. The effective tags were subjected to denoising using DADA2 within QIIME2 software (Version QIIME2-202006) to generate the initial amplicon sequence variations (ASVs). Subsequently, ASVs with an abundance of less than five were filtered out. Species annotation of the obtained ASVs was performed using classify-sklearn within QIIME2 software by comparing with the Silva database (v138.1). The absolute abundance of ASVs was normalized using a standard sequence number corresponding to the sample with the least sequences. Subsequent analyses of α- and β-diversities were all performed based on the output normalized data. The Shannon and Chao1 indices were used to determine the α-diversity. The PCoA and NMDS analyses were conducted to visualize β-diversity by using R software (v4.3.2). The gene function of the acquired OTUs was predicted using PICRUSt2 software (v2.1.2-b). The network analysis was performed using R with the Hmisc package; *P*-value was adjusted using the Benjamini-Hochberg method.

### Statistics

For the comparison of blood indicators and liver copper, the Shapiro-Wilk test was first used to determine the distribution of the data from each group. One-way analysis of variance was used to analyze the differences between any two data sets that followed a normal distribution, and the Tukey test was used for pairwise comparison. In cases where the data did not meet the criteria for normal distribution, a non-parametric test (Mann–Whitney U test or Kruskal–Wallis test) was used to analyze the differences between groups. To compare the abundance of bacterial taxa between the two groups, the Wilcoxon rank sum test was employed in conjunction with the Benjamini–Hochberg method. The Spearman correlation test was used to analyze the correlation between variables. The statistical significance was observed when *P* < 0.05.

## Data Availability

The study’s original findings are accessible to the public. The data is available in NCBI Sequence Read Archive (SRA) with BioProject ID PRJNA1014229.

## SUPPLEMENTAL MATERIAL

Fig. S1 spectrum.00573-24-s0001.tif DPA and Akk treatment alleviates gut dysbiosis in WD mice (genus level).

Supplemental legend spectrum.00573-24-s0002.docx Legend for Fig. S1.

ASM does not own the copyrights to Supplemental Material that may be linked to, or accessed through, an article. The authors have granted ASM a non-exclusive, world-wide license to publish the Supplemental Material files. Please contact the corresponding author directly for reuse.
