# Supplementary material for: Gut Akkermansia enhances liver protection and facilitates copper removal during D-penicillamine treatment in a Wilson’s disease model
Source: Microbiol Spectr. 2025 Mar 31;13(5):e00573-24. doi: 10.1128/spectrum.00573-24 (PMC12054026; doi:10.1128/spectrum.00573-24)
Supplement: Supplemental legend — Legend for Fig. S1. [file spectrum.00573-24-s0002.docx]

**Figure legends**

**Figure. S1** DPA and Akk treatment alleviates gut dysbiosis in WD mice (genus level)
